# Barriers to Middle-Aged Women’s Mental Health: A Qualitative Study

**DOI:** 10.5812/ircmj.18882

**Published:** 2014-06-05

**Authors:** Khadijeh Sharifi, Monireh Anoosheh, Mahshid Foroughan, Anushirvan Kazemnejad

**Affiliations:** 1 Department of Nursing, Tarbiat Modares University, Tehran, IR Iran; 2 Department of Gerontology, Aging Research Center, University of Social Welfare and Rehabilitation Sciences, Tehran, IR Iran; 3 Department of Biostatistics, Tarbiat Modares University, Tehran, IR Iran

**Keywords:** Women, Mental Health, Middle Aged, Qualitative Research

## Abstract

**Background:**

Middle-aged women encounter some barriers to their mental health, putting them at great risk for developing mental disorders.

**Objectives:**

The aim of this study was to explore barriers to middle-aged women’s mental health.

**Materials and Methods:**

This was a qualitative content analysis study conducted in 2013 in Kashan, Iran. A purposive, maximum variation sample of 23 middle-aged women was recruited to the study. Data were collected by conducting semi-structured individual interviews. We employed the conventional qualitative content analysis approach for data analysis.

**Results:**

Barriers to middle-aged women’s mental health fell into two main themes including ‘increased life concerns’ and ‘physical and psychological tensions’. The two sub-categories of the first theme included having mental concerns and increased burden of roles. The second main theme also consisted of two categories including perceived undesirable physical changes and perceived undesirable psychological changes.

**Conclusions:**

Experiences of middle-aged women showed that culturally appropriate interventions to alleviate the concerns of life, physical and mental stress is essential to preserve stability of mental health.

## 1. Background

Middle-age period is an important phase of life that lies between being young and old (1, 2). Because of different biological, mental, social, and psychological alterations, middle-aged people are at greater risk for developing mental problems (3).

Middle-aged women, compared to their male counterparts, experience more age-related health changes and complications. The most serious incident of the middle-age period is menopause and subsequent infertility, which negatively affects women’s self-concept and self-esteem (3, 4). Physical outcomes of menopause are severely affecting quality of life in middle-aged women (5). In this period women face with various problems such as; retirement, multiplicity of roles and responsibilities, financial problems, empty nest syndrome, weight gain, and changes in appearance (2, 6, 7). In recent years, our society is in transition from the extended family to the core family. This change in family structure and many problems in this period of life endanger middle-aged women’s mental health and led to an increased number of patients with mental disorders (8).

The findings of a study conducted by Ahmadvand et al. in Kashan, Iran, revealed that 35.5% of women and 35.8% of middle aged people are affected with mental disorders and the findings of a study conducted by Mohammadi et al. in Iran revealed that mood and anxiety disorders in women and middle age people was more than men and older ages, respectively (9, 10).

Singh and Singh in India also reported that 54% of women participating in their study experienced moderate to high stress, 32% moderate stress, 44% moderate depression and 80% social dysfunction (3). The results of study of Wang *et al.* in Taiwan showed a 38.7% prevalence of depression in middle-aged women (45-60 years old) (11). According to above items listed, addressing barriers to middle-aged women’s mental health is necessary.

## 2. Objectives

Given the vulnerability of middle-aged women to different health problems, this study was conducted to explore barriers to middle-aged women’s mental health.

## 3. Materials and Methods

This was a qualitative content analysis study conducted in 2013. Qualitative content analysis is a systematic approach that provides a detailed description of a deeper insight into the intended phenomenon. This approach is useful for exploring people’s attitudes, perceptions, and interpretations about life experiences (12-14).

A gradual purposive sample of 23 middle-aged women was recruited into the study. The inclusion criteria were having an age of 40-65 years (7), living in Kashan, Iran, no confirmed previous psychiatric diagnosis, and having the ability to clearly speak and understand Persian. The only exclusion criterum was lack of desire to continue cooperation with the study. To include a wide range of viewpoints, we strived to recruit a maximum variation sample in terms of certain characteristics such as age, number and gender of children, marital and employment status, income level, and menopausal occurrence. Demographic characteristics of participants are shown in Table 1.

We collected the study data by conducting semi-structured individual interviews. Interviewer was a PhD student of nursing with 15 years experiences of counseling. The main interview question included but not limited to “During middle age period, what factors have negatively affected your mental health? What factors in middle-age have adverse effect on your comfort?” We also used probing questions to delve into the participants’ experiences. The next questions were adjusted on the basis of participants' responses.

**Table 1.** Demographic Characteristics of Participants

Variable	Frequency. (%)
Age, y	
40-44	4 (17.4)
45-49	6 (26.1)
50-54	5 (21.7)
55-59	4 (17.4)
60-65	4 (17.4)
**Education**	
Illiteracy or primary school	11 (47.8)
Secondary or high school	5 (21.7)
University	7 (30.5)
**Marital status**	
Single	1 (4.33)
Married	20 (87)
Dead wife	1 (4.34)
Divorcee	1 (4.34)
**Job status**	
Employed	9 (39.1)
Housekeeper	14 (60.9)
**Total**	23 (100)

Interviews were arranged according to the participants’ preferences regarding location and time of interview. The length of the interviews ranged from 40 to 90 minutes. Data collection was continued until reaching data saturation, i.e. when no new data was obtained from the old and new interviews. Accordingly, we held 23 interviews with 23 middle-aged women. All of the interviews were recorded after getting informed consent.

Data analysis was conducted simultaneously with data collection. For data analysis manually, we went through the following eight-step process of the conventional qualitative content analysis approach: 1. Sorting the data; 2. Deciding on the unit of analysis; 3. Planning for developing the generated codes and categories; 4. Coding the excerpts; 5. Coding the whole text; 6. Achieving consistency in coding; 7. Concluding about the coded data; and 8. Reporting methods and findings (12, 14).

Accordingly, immediately after each interview, we transcribed it verbatim and read the transcript several times to obtain a general sense of the content and to immerse in the data. Then, we (3 coders) identified and coded the meaning units. We constantly compared the identified meaning units and the generated codes with each other and categorized them according to their similarities and differences. Sub-categories and categories were, in turn, compared with each other and categorized into more abstract sub-themes and themes (12, 13, 15). Table 2 show the development of ‘irritating concerns’ theme and its related meaning units, codes, sub-categories, and categories.

We employed different techniques for enhancing the rigor of the study. It's used from transferability, dependability, credibility and conformability to ensure the trust worthiness of data. The credibility of the study findings was established by using the member-checking technique. The codes that did not reflect the participants’ view-points were revised according to their comments. Moreover, two nursing faculty members appraised and approved the generated codes and categories. Prolonged engagement in data collection and analysis as well as analyzing data simultaneously with data collection helped us enhancing the dependability of the study findings. We also strived to recruit a maximum variation sample to increase the credibility and transferability of the findings. Limitation of this study like other qualitative studies was inability to generalized finding to the target population.

The Ethics Committee of Tarbiat Modares University approved the study (with code D-52/3785). We provided the study participants with information about the aim and the process of the study and ensured them that their personal information will be managed confidentially and reported anonymously. Appropriate environment was provided during the interview and results were given if desired. The study participants were free to participate in or withdraw from the study. We obtained a written informed consent from each participant.

**Table 2.** The Development of ‘Irritating Concerns’ Theme

Meaning Units	Codes	Sub-Categories	Categories
**Thinking about the past as well as my past mistakes put me under considerable pressure**	Thinking about past mistakes	Preoccupation with the past	Having mental concerns
**I am always thinking about my children’s future**	Preoccupation with children’s prospect	Preoccupation with future	
**I’m worried about developing Alzheimer**	Preoccupation with the risk of developing Alzheimer	Preoccupation with personal health	
**My children’s bachelorhood annoys me and occupies my mind**	Preoccupation and annoyance with children’s bachelorhood	Preoccupation with family problems	
**Financial problems also constantly worry me**	Worry and preoccupation with financial problems	Preoccupation with financial problems	
**I have to do my own and children's housework**	Doing one’s own and married children’s housework	Increased burden of maternal role	Increased burden of roles
**Taking care of my ill parents during several years was really arduous**	Tiresome care for disabled parents	Increased burden of filial roles	
**My husband does not feel financially responsible toward me; I do shopping and heavy housework on my own**	Feeling sad over husband’s financial irresponsibility as well as being compelled to do heavy works alone	Increased burden of spousal roles	

## 4. Results

Totally, 742 primary codes were generated from the transcripts. All these codes fell into two main themes, including ‘increased life concerns’ and ‘physical and psychological tensions’ with the related categories and sub-categories.

### 4.1. First Theme: Increased Life Concerns

Most of the participants mentioned that they have felt many concerns during their middle age period. The increased life concern theme consisted of two main categories including ‘having mental concerns, and ‘increased burden of roles’.

### 4.2. Having Mental Concerns

While going through the middle ages of life, women’s children get married and leave parent’s home. On the other hand, women’s parents grow older and become increasingly dependent. Moreover, middle-aged women experience physical health problems and become increasingly worried about their own future. Our participating middle-aged women complained of growing preoccupation and considerable inconvenience, because of such alterations in their lives. The sub-categories of the mental concerns theme included.

#### 4.2.1. Preoccupation With the Past

Most of our participants found recalling and reviewing memories and difficulties of the past irritating and referred to them as barriers to their mental health. A 43-year-old woman noted, “Thinking about the past events as well as my past mistakes put me under considerable pressure” (P. 14).

#### 4.2.2. Preoccupation With Future

Our participating women were also preoccupied with future events particularly their children’s prospect. A 42-year-old woman mentioned, “I am always thinking about my children’s future” (P. 15).

#### 4.2.3. Preoccupation With Personal Health

Most of the participants were experiencing different problems with their own physical health. Accordingly, they were extremely worried about becoming physically unable and dependent later in life. A 53-year-old woman stated, “Compared to the past, I’m more forgetful now. I’m worried about developing Alzheimer” (P. 16).

#### 4.2.4. Preoccupation With Family Problems

Preoccupation with family problems was another concern for our participants. A 58-year-old woman said, “My children’s bachelorhood tends to annoy me and occupy my mind. I’m extremely worried and anxious” (P. 6).

#### 4.2.5. Preoccupation With Financial Problems

Lack of financial security during the middle age period also had damaged our participants’ self-worth, self-esteem, and hence, mental health. A 43-year-old woman noted, “Financial problems also occupy my mind, worry me, and put me under considerable pressure” (P. 14).

### 4.3. Increased Burden of Roles

Most of our participants were experiencing increased burden of roles and responsibilities and considered the increased burden of roles as troubling and tiring. The sub-categories of this category included:

#### 4.3.1. Increased Burden of Maternal Role

Beside maternal and spousal roles, our participating women also needed to assume and fulfill their added roles as mother-in-law and grandmother roles during their middle ages. These roles burdened our participants with heavy responsibilities. A 61-year-old woman noted, “I have to do my own and children's housework. I’m under considerable pressure” (P. 10).

#### 4.3.2. Increased Burden of Filial Roles

Most of our participants had shouldered the responsibility of providing care to their elderly parents. A 61-year-old woman said, “Taking care of my ill parents during several years was really arduous” (P. 10).

#### 4.3.3. Increased Burden of Spousal Roles

Our participants also complained of increased burden of spousal roles. A 46-year-old participant stated, “My husband does not feel financially responsible toward me; I do shopping and heavy housework on my own” (P. 7).

### 4.4. Second Theme: Physical and Psychological Tensions

Middle-aged women usually undergo considerable changes in their physical and psychological health which cause them great emotional stress. The physical and psychological tensions theme consisted of two categories including ‘perceived undesirable physical changes’ and ‘perceived undesirable psychological changes’.

#### 4.4.1. Perceived Undesirable Physical Changes

Our participants were suffering from negative effects of undesirable physical changes on their self-confidence, self-worth, and sense of well-being. The four sub-categories of this category included:

##### 4.4.1.1. Undesirable Changes in Appearance

Our participants referred to changes in appearance as distressing experiences of middle age and signs of aging. A 51-year-old participant said, “The first and the most prominent signs of aging were in my face and appearance” (P. 11).

##### 4.4.1.2. Declined Energy

Midlife women complained from decreased physical ability. A 50-year old woman expressed, “In the past, my body was very strong, no work did bother me at all, but now I feel tired soon” (P. 1).

##### 4.4.1.3. Increased Physical Disease and Problems

Our participants believed that middle age is the period of increased risk for developing different types of diseases. A 59-year-old woman expressed, “I have been experiencing high blood pressure, and my blood lipid and glucose have increased” (P. 9).

##### 4.4.1.4. Menopausal Negative Changes

Our participants noted that menopausal symptoms, particularly vasomotor ones, really irritated them. A 55-year-old woman mentioned, “After menopause, I experienced flushing and excessive perspiration. I’ve had no relationship with my husband” (P. 2).

#### 4.4.2. Perceived Undesirable Psychological Changes

These changes had caused participants' anxiety and depression. The four sub-categories of this category included:

##### 4.4.2.1. Declined Mood

According to our participants, termination of the youth period, increased burden of roles, and development of different age-related problems (such as menopause) prevented them from enjoying life and threatened their mental health. A 46-year-old participant noted, “I enjoy life very much less than before; I used to be happier” (P. 7).

##### 4.4.2.2. Decline in Mental Functions

Our participants complained of decline in mental functions, slow learning and memory problem. A 62-year old woman mentioned, “My memory has been poor” (P. 8).

##### 4.4.2.3. Increased Tensions

Most of our participants noted of increased tension in job and family environment. A 47-year-old participant said, “I’m suffering from occupational stress, authorities don’t understand us” (P. 4). A 59-year-old woman mentioned, “One of my brothers suffers from cancer. One of my sons wants to get married but he cannot find a job. My oldest son has detached himself from me” (P. 9).

##### 4.4.2.4. Dissatisfaction with Aging

Most of our participants felt dissatisfied with aging and age-related physical and mental decline. A 46-year-old participant stated, “Middle age is a nasty time period; a period of mental and physical decline; I’m not satisfied with it” (P. 7).

## 5. Discussion

This study explored the barriers to middle-aged women’s mental health experiences. The findings of the study revealed that barriers to middle-aged women’s mental health fall into two main themes including ‘increased life concerns’ and “physical and psychological tensions”. Increased burden of roles and feeling concerned about past, present, and future were among the most important irritating concerns experienced by our participants. The study participants highlighted the important role of increased burden of maternal, filial, and spousal roles in undermining their mental health. Hye-Sook et al. also reported the increased burden of roles and responsibilities among Korean middle-aged women. They found that assuming the responsibility for providing care to people of different age groups, e.g. parents and children, was stressful for the participating women (16). Hildingh et al. also noted that the multiplicity of roles greatly undermines women’s health (17). Age-related undesirable changes such as children leaving home and its subsequent empty nest syndrome, husband’s health problems and death, retirement and its subsequent limited job security, as well as the necessity for providing care to elderly parents increase middle-aged women’s roles and responsibilities. Role changes happening during middle age not only undermines women’s mental health, but also affects their social functioning (18, 19).

The study participants were also extremely concerned about past, present, and future. During middle ages, women face many stressors such as family conflicts, self and family health problems, and childrearing difficulties (16). These stressors can cause great concern for middle-aged women and damage their mental health.

The second theme of the study- physical and psychological tensions -consisted of two categories including perceived undesirable physical changes and perceived undesirable psychological changes. A sub-category of the perceived undesirable physical changes category was undesirable changes in appearance. Cifcili et al. also found that Turkish middle-aged women suffered from disturbed body image, self-hate, and a sense of ugliness (20). Age-related changes in appearance such as facial wrinkles, appearance of gray hairs, decreased visual acuity, weight gain, and decreased physical ability can undermine women’s self-confidence, self-worth, and well-being and hence, caused them different mental health problems such as depression (3, 21). Physical decline like energy decline, and occurrence of physical disease and problems was another barrier to our participants’ mental health. Ayranci reported that in middle-aged women, the risk for developing at least one chronic disease is 83.2% (7). Shojaeyan et al. also found that physical health problems are highly prevalent among middle-aged women (6). Ayranci and Shojaeyan et al. reported that middle-aged women are at greater risk for developing physical health problems (6, 7). Given the age-related impairment of physical ability, middle-aged women inevitably experience difficulties in maintaining and improving their health.

Another sub-category of the perceived undesirable physical changes category was menopausal negative changes. Ayranci found that the prevalence rates of physical problems such as flushing; back pain, headache, and fatigue among Turkish middle-age menopausal women were respectively 96.5%, 95%, 91.7%, and 34.9% (7). Chuni and Sreeramareddy also reported that most of Nepalese middle-age menopausal women experience sleep disorders (78%), physical and mental fatigue (73.5%), flushing (69.7%), musculoskeletal problems (68.6%), and vaginal dryness (61.6%) (4).

Mood decline was one of the sub categories of perceived common undesirable psychological changes that had happened to our participants. Ahmadvand et al. found that compared to other age groups, mental disorders were more prevalent among Iranian middle-aged women (9). Singh and Singh also reported that most of Indian middle-aged women participating in their study experienced unpleasant psychological changes as well as moderate to severe stress (3). Based on the findings, middle aged women complained of decline in mental function. Rassoli et al. in Ilam, Iran, found that %39.2 of middle age women suffer from memory problems (22). Our participants also referred to employment-related tensions as a major barrier to their mental health. Hye-Sook et al. found that middle-aged women suffered from high levels of psychological stress (16). Family problems such as conflicts and diseases as well as uncertainty over future can play an important role in causing stress.

The middle-aged women participated in our study also noted that dissatisfaction with aging and becoming middle-aged, threatened their mental health. Age-related appearance changes, physical decline, and menopause give middle-aged women a sense of senility. Having a sense of wasting youth also can contribute to dissatisfaction with aging.

The findings of this study lead to a better understanding of mental health barriers in middle-aged women. It is hoped that healthcare professionals, particularly nurses, improve community mental health and take effective steps with specific and culturally appropriate planning in order to reduce the concerns of life and physical and psychological tensions of middle-aged women.
